# A Rare Case of Disseminated Histoplasmosis With Hemophagocytic Syndrome in a Patient With Diabetes Mellitus: A Case Report

**DOI:** 10.7759/cureus.36333

**Published:** 2023-03-18

**Authors:** Harshit Gupta, Pradeep Yadav KL, Manjunath Totaganti, Ravi Kant, Yumkham Monica Devi

**Affiliations:** 1 Internal Medicine, All India Institute of Medical Sciences, Rishikesh, IND; 2 Internal Medicine, Sri Siddhartha Medical College, Tumkur, IND

**Keywords:** amphotericin b, fungal infections, antifungal, pancytopenia, immunocompetent, hemophagocytic syndrome, histoplasmosis, diabetes mellitus

## Abstract

We report a case of Histoplasma-associated hemophagocytic syndrome in a diabetes mellitus patient. The patient presented with a fever, cough, and an ulcer on the tongue. The biopsy confirmed the diagnosis of histoplasmosis from the tongue ulcer. Other investigations revealed normal clusters of differentiation 4 (CD4) count and increased hemoglobin A1c (HbA1c) and lactate dehydrogenase (LDH) levels. The patient was diagnosed with hemophagocytic syndrome secondary to Histoplasma after fulfilling the hemophagocytic lymphohistiocytosis (HLH)-2004 criteria required for diagnosis, including fever (with peak temperatures of >38.5° C), splenomegaly, cytopenia affecting two cell lineages in peripheral blood, hypertriglyceridemia (fasting triglycerides >265 mg/dL), and hemophagocytosis in the bone marrow biopsy. The patient was started on injection amphotericin B with remarkable improvement.

## Introduction

Histoplasmosis is caused by a dimorphic fungus, Histoplasma capsulatum, which is rare in India but endemic in the western region, the Gangetic Plains, and small areas of West Bengal [1,2,3]. It prefers the reticuloendothelial system, leading to hepatosplenomegaly, a prominent feature of disseminated histoplasmosis [4]. It is primarily a pulmonary disease but can progress to disseminated disease in immunocompromised patients, although some disseminated histoplasmosis cases are reported in immunocompetent patients [5,6]. The authors describe a case of hemophagocytic syndrome secondary to disseminated histoplasmosis in a diabetic mellitus patient with uncontrollable sugar levels (hemoglobin A1c (HbA1c) of 8.1%).

## Case presentation

A 62-year-old patient, a resident of the sub-Himalayan region, has been a known diabetic on oral hypoglycemic medication for the last three years. He presented with an ulcer over the tongue for the last eight months and a high-grade intermittent fever for six weeks. It was associated with coughing with expectoration, decreased appetite, and weight loss. There was no associated burning micturition, cough, expectoration, headache, malaise, abdominal pain, vomiting, loose stools, hair loss, or photosensitivity. On examination, the patient was febrile (100°F) and icteric. Systemic examination revealed hepatosplenomegaly and coarse crepitations in the bilateral infrascapular region.

Routine investigation revealed anaemia, thrombocytopenia, direct hyperbilirubinemia, an increased alkaline phosphatase level, and increased lactate dehydrogenase (LDH) and triglyceride levels (as shown in Table 1).

**Table 1:** Results of complete blood count, liver function test, and renal function test PP: postprandial; LDL: low-density lipoprotein; LDH: lactate dehydrogenase

Investigations	Reference Range (adults, this hospital)	Day One
Haemoglobin (g/dl)	13.5-17.5	8.2
White cell count (per μl)	4500–11,000	8874
Differential count (per μl)		
Neutrophils	1800–7700	7823
Lymphocytes	1000–4800	612.3
Monocytes	200–1200	263
Eosinophils	0–900	70
Basophils	0–300	100
Platelets (per μl)	150,000–400,000	38000
Liver function tests		
Aspartate aminotransferase (U/L)	5-40	37.2
Alanine aminotransferase (U/L)	5-45	65.7
Total bilirubin (mg/dL)	0.2-1.1	1.4
Direct bilirubin (mg/dL)	<0.20	0.74
Alkaline phosphatase (U/L)	<240	296.8
Serum total protein (g/dL)	6.4-8.1	6.9
Serum albumin (g/dL)	3.2-4.6	3.6
Renal function test		
Sodium (mmol/L)	135–145	130.5
Potassium (mmol/L)	3.4–5.0	4.1
Chloride (mmol/L)	100–108	91.9
Urea nitrogen (mg/dL)	8–25	43.9
Creatinine (mg/dL)	0.60–1.50	0.87
Glucose PP (mg/dL)	70–140	213
Cholesterol (mg/L)	<200	89.6
Triglycerides (mg/L)	<150	298.4
LDL (mg/dL)	<30	59.68
LDH (U/L)	240-480 U/L	861.8

**Figure 1:**
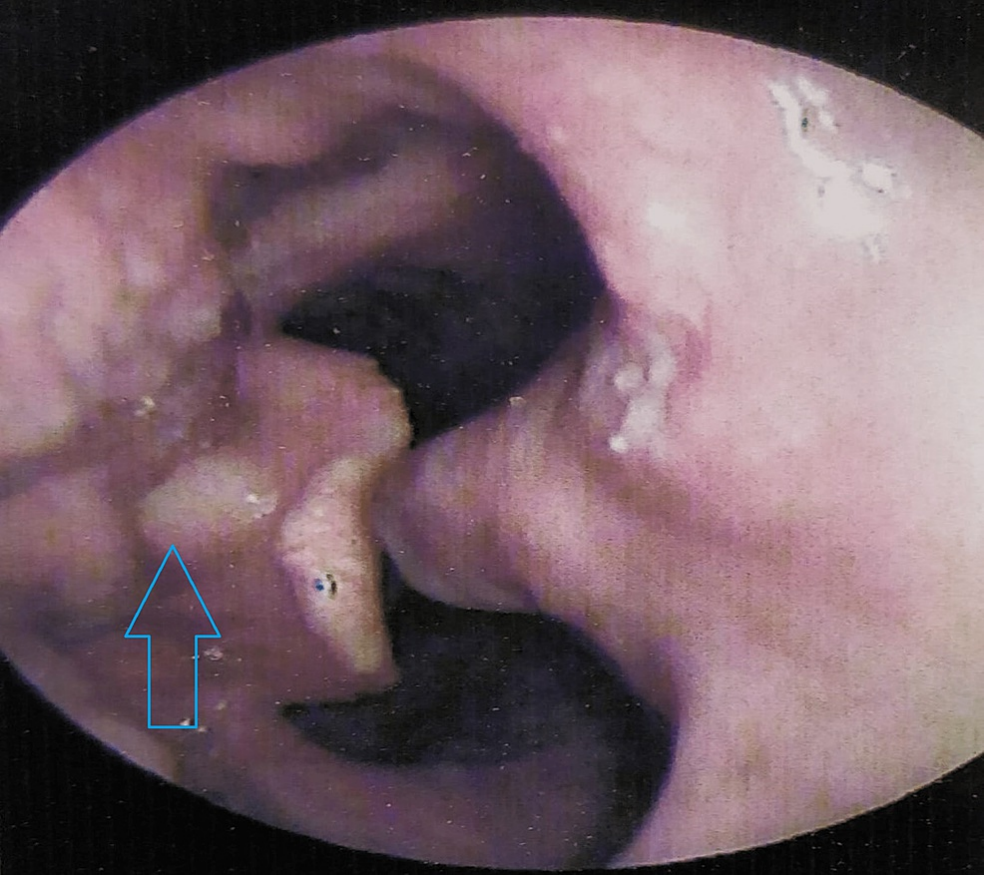
Endoscopy showing multiple ulcers on the base of the tongue and pharynx (marked by a blue arrow)

Endoscopy revealed multiple ulcers on the base of the tongue and pharynx (as shown in Figure 1). The histopathology report of ulcer scrapings from the tongue revealed granulomatous inflammation consisting of epithelioid histiocytes and giant cells. Along with these, there were round to oval organisms surrounded by clear spaces with budding forms within the macrophages, which stained positive for Gomori methenamine silver, suggestive of Histoplasma.

**Figure 2:**
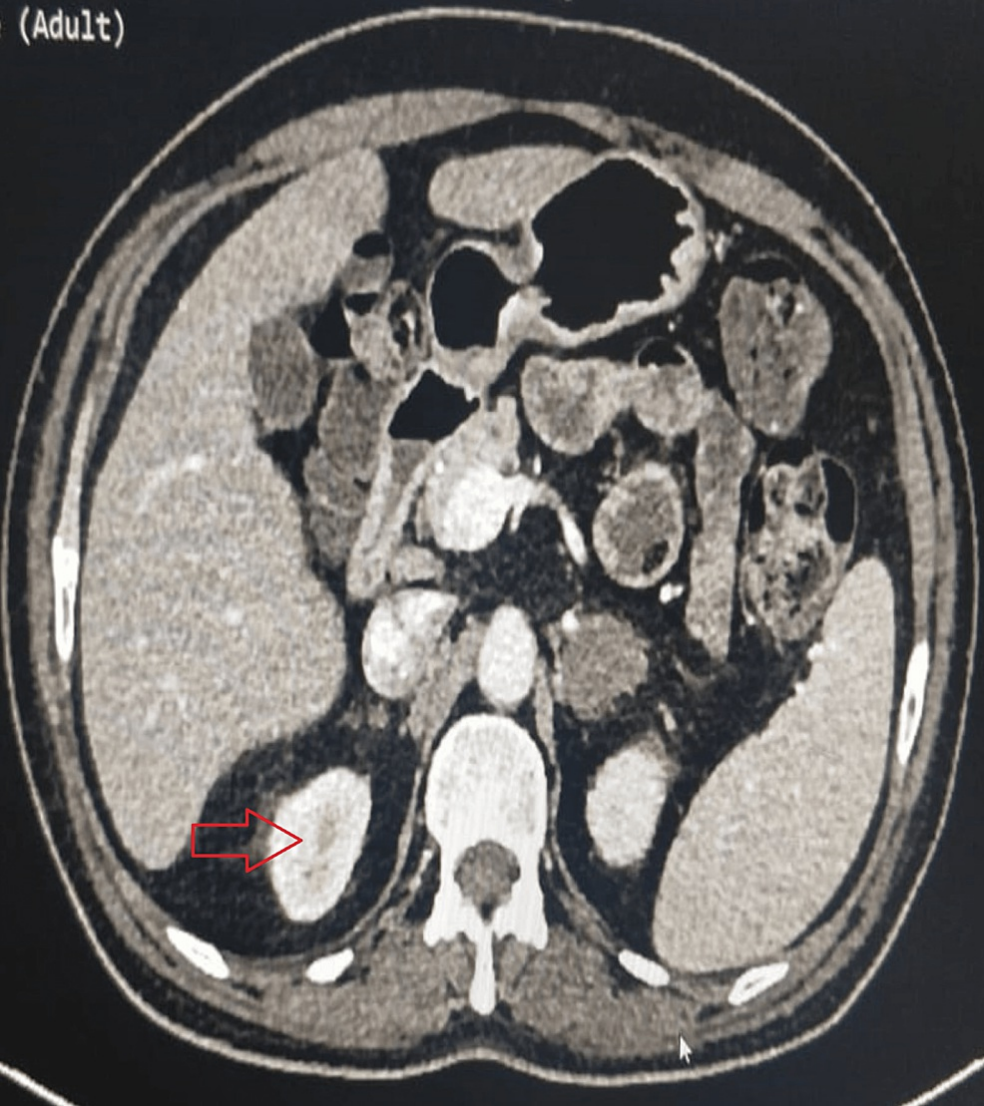
The CECT of the abdomen showing a non-enhancing lesion in the adrenal gland (marked by a red arrow)

High-dose contrast-enhanced computed tomography (CECT) of the thorax revealed an enhancing nodule in the superior segment of the left lower lobe and fibronodular changes with adjacent pleural thickening noted in bilateral lung apices. In contrast, the CECT of the abdomen revealed hypodense, non-enhancing lesions in both adrenal glands along with mild surrounding fat stranding and hepatosplenomegaly (as shown in Figure 2).

The patient's clusters of differentiation 4 (CD4) T-cell count was within the normal range, but his HbA1c was 8.1%, suggesting uncontrollable diabetes mellitus. A bone marrow biopsy done in this patient revealed hemophagocytosis along with a reduced platelet count. A urine Histoplasma antigen was also done, which came out positive. A diagnosis of disseminated histoplasmosis with secondary hemophagocytic syndrome was made, and the patient was started on injection amphotericin-B. On the fourth day of treatment, the patient started improving clinically. After two weeks of amphotericin-B therapy, he was shifted to oral itraconazole, 200mg twice a day for 12 months.

## Discussion

Histoplasmosis caused by Histoplasma capsulatum usually occurs after exposure to bird and bat guano [6]. After inhalation of microconidia, it may cause pulmonary involvement, presenting as pneumonia, or may remain asymptomatic [7]. However, immunodeficiency patients can develop a disseminated disease involving the bone marrow, adrenal glands, gastrointestinal system, and reticuloendothelial system [7,8].

In this study, a known case of diabetes mellitus treated with an oral hypoglycaemic agent presented with fever, non-productive cough, a tongue ulcer, and weight loss. Investigation revealed anaemia, thrombocytopenia, increased LDH and triglyceride levels, direct hyperbilirubinemia, along with adrenal and bone marrow involvement. Tongue scraping of the ulcer confirmed the histoplasmosis. A diagnosis of hemophagocytic syndrome secondary to disseminated histoplasmosis was made. The patient was given an injection of amphotericin B. The patient started to improve clinically by the fourth day of treatment, and on the 14th day of treatment, he was shifted to oral itraconazole 200mg twice a day and was discharged. The patient was followed up for a year, and oral itraconazole was continued for the same duration. A bone marrow biopsy was done post-treatment, which returned normal.

A hemophagocytic syndrome is a hyperinflammatory state responsible for fatality if not recognized promptly and treated [9]. A study from Chicago has reported six patients with hemophagocytic syndrome secondary to disseminated histoplasmosis in an AIDS patient [10]. In addition to AIDS, these cases are also reported in renal transplant and chronic lymphoid leukaemia patients [11,12]. In previously reported cases, it has been hypothesized that the NK cell becomes defective in acquired immunodeficiency patients. This leads to impaired clearance of the activated T-cells and activated histiocytes, leading to a cytokine storm and hyperinflammatory state observed in hemophagocytic syndrome [13].

In disseminated histoplasmosis, it is recommended to give treatment with an injection of amphotericin B for a duration of two weeks. The patient normally starts responding to treatment clinically within this time period. Post this, the patient is shifted to oral azoles, which are continued for a duration of six to 18 months. Among oral azoles, itraconazole is the preferred drug. Fluconazole is the second-line azole, which is only used when itraconazole is intolerable [14].

## Conclusions

In conclusion, Histoplasma capsulatum, which primarily leads to pulmonary disease and disseminated disease in immunocompromised patients, can also lead to disseminated disease in diabetes mellitus patients, along with the rare complication of secondary hemophagocytic syndrome, which has been previously reported in AIDS, renal transplant, and chronic lymphoid leukaemia patients suffering with histoplasmosis. A bone marrow biopsy can help confirm hemophagocytic syndrome in these patients with suspected disseminated histoplasmosis.
